# Do coronary stents suffer long‐term deterioration after repeated intracoronary lithotripsy for rebel underexpansion treatment?

**DOI:** 10.1002/ccr3.6547

**Published:** 2022-11-15

**Authors:** Jose Valencia, Marta Herrero‐Brocal, Fernando Torres‐Mezcua, Javier Pineda, Juan Miguel Ruiz‐Nodar

**Affiliations:** ^1^ Servicio de Cardiología Hospital General Universitario de Alicante Alicante Spain; ^2^ Unidad de Hemodinámica y Cardiología Intervencionista Hospital General Universitario de Alicante Alicante Spain

**Keywords:** coronary angioplasty, intracoronary lithotripsy, optical coherence tomography, restenosis

## Abstract

Coronary intravascular lithotripsy (IVL) is the latest developed technique available for stent underexpansion treatment, although it is unclear if this therapy causes stent structure damage. We present the case of a patient with severe, refractory stent underexpansion after primary angioplasty, which was resolved with a double session of IVL. Elective angiographic and optical coherence tomography (OCT) follow‐up was performed 1 year after the procedure, which demonstrated the absence of any damage in the stent platform. Paradoxically, the study revealed a critical restenotic lesion in an area distant from the one of interest. Review of the first OCT after the primary procedure revealed 78% underexpansion in that area, which went by unnoticed and could be the cause of restenosis. Repeated IVL therapy may be helpful in cases of rebel stent underexpansion, and it conveys the impression of being safe in the long term in relation to the integrity and effectiveness of the drug‐eluting coronary stents.

## Graphical abstract

Repeated IVL therapy may be helpful in cases of rebel stent underexpansion, and it conveys the impression of being safe in the long term in relation to the integrity and effectiveness of drug‐eluting coronary stents.

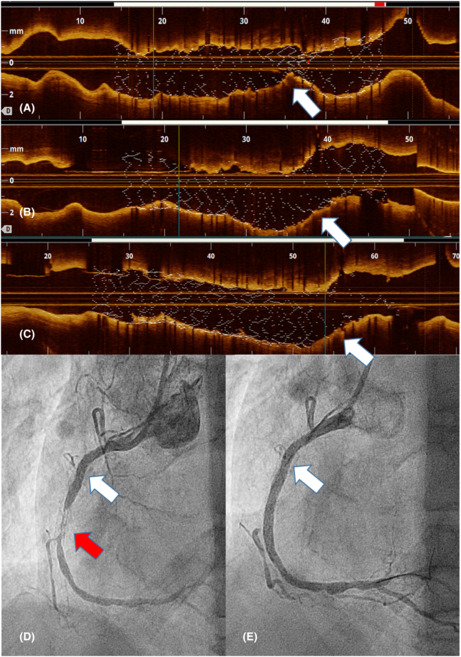

## 1 INTRODUCTION

Stent underexpansion, due to the presence of plaques with extensive areas of calcification, is an unusual but difficult‐to‐manage complication. Coronary intravascular lithotripsy (IVL) is the latest developed technique available to interventional cardiologists for its treatment, although it is not effective in all cases.^1^ In addition, it is unclear if this therapy causes damage to the structure of the metal framework of the stent or to the polymer responsible for transporting the antiproliferative drug of the latest generation drug‐eluting stents.^2^

## 2 REPORT

We present the case of a patient with severe and refractory stent underexpansion with a Cr‐Pt platform after primary angioplasty (only 36% final expansion achieved) who required a double session of intracoronary lithotripsy for its entire resolution. Both sessions were 72 h apart and the maximum 160 pulses allowed were administered (80 pulses for each of the 3‐ and 3.5‐mm lithotripsy balloons used).^3^

**FIGURE 1:**
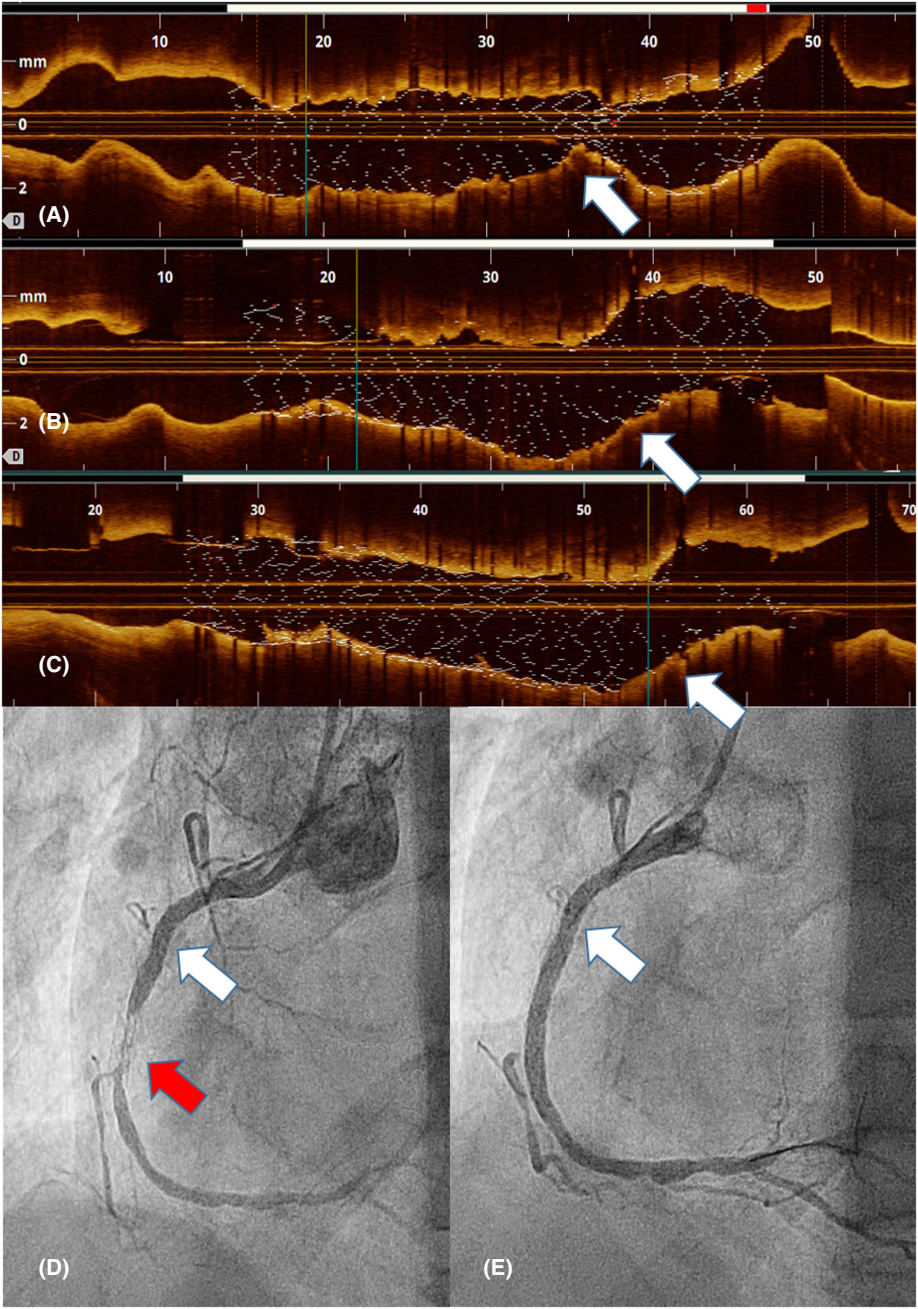
Upper part: Longitudinal reconstruction of the vessel by optical coherence tomography. (A) After the first cycle of lithotripsy, (B) after the second cycle, (C) results at 1 year. White arrows indicate the lithotripsy treatment area. Lower part: (D) Angiography of the right coronary artery 1 year after the procedure. The white arrow indicates the area treated with lithotripsy and the red arrow the area where restenosis appeared. (E) Final results after a new angioplasty

**FIGURE 2:**
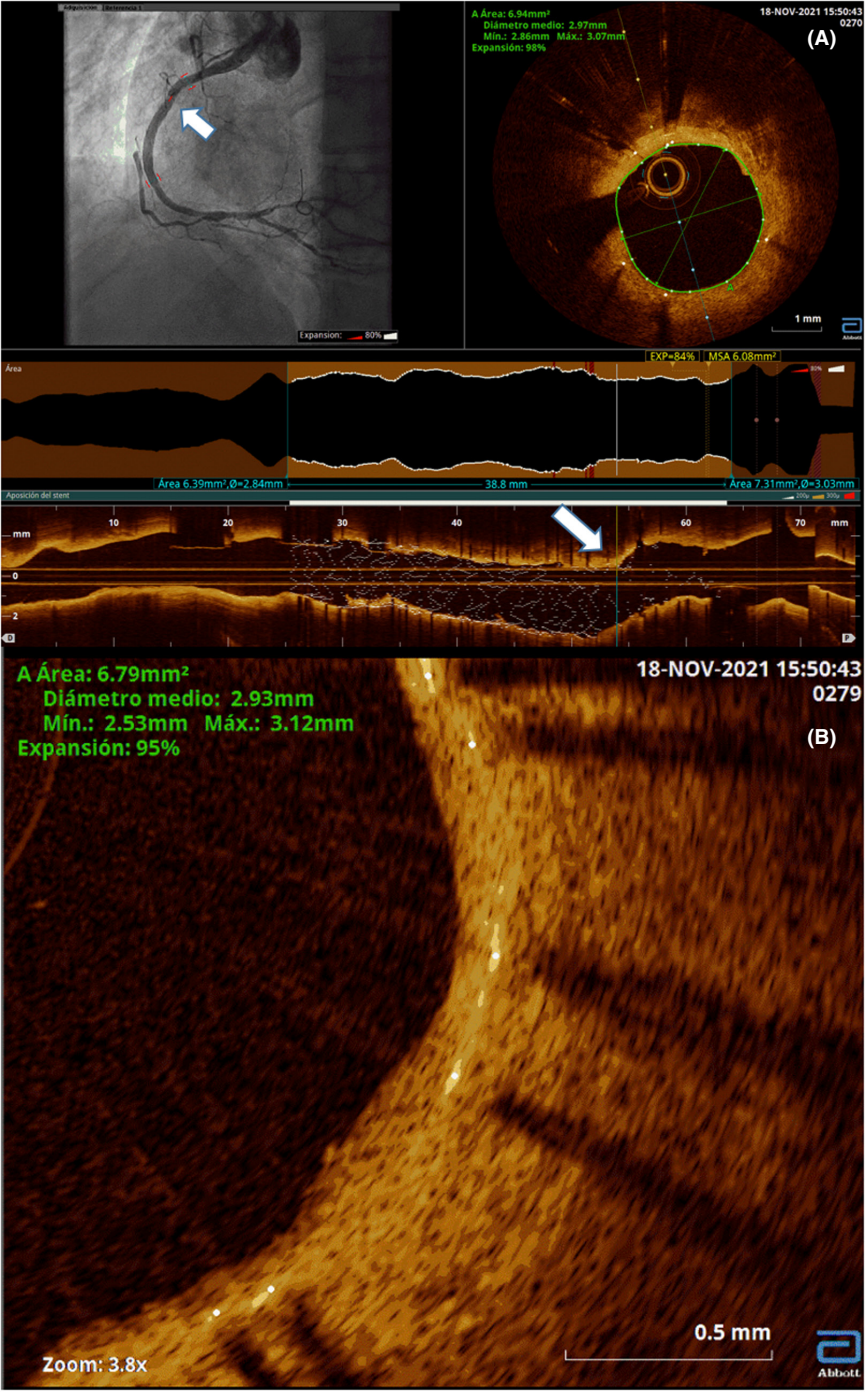
**(**A) One‐year follow‐up, optical coherence tomography sagittal slice at the level of the lithotripsy treatment area (white arrows) with angiographic co‐registration. (B) Zooming in on this area shows in detail the endothelialization of the strut and the slight intimal hyperplasia.

Elective angiographic and optical coherence tomography (OCT) follow‐up was performed 1 year after the procedure (Figures 1 and 2). It was possible to verify how the success of the procedure was sustained in the area that received the double dose of maximum lithotripsy therapy, and the stent structure was maintained, without collapse at any level, with complete endothelialization of its struts and minimal restenosis due to intimal benign features proliferation. The aggressive lithotripsy in our case did not lead to impaired integrity and effectiveness of the drug‐eluting coronary stents over a 1‐year period.

Paradoxically, the study revealed a critical restenotic lesion in an area distant from the one under follow‐up. Review of the first OCT after the primary procedure revealed 78% underexpansion in the area that went unnoticed and could be the cause of restenosis. This restenotic lesion showed characteristics of a neoatherosclerotic condition according to the OCT findings. Hence, it is logical to think that patient's outcome, if the underexpansion under analysis was not resolved, could have led to a much more aggressive proliferative phenomenon at that level, with a probable first manifestation as acute coronary syndrome. This lesion was satisfactorily resolved by performing a new scoring balloon angioplasty, followed by the implantation of a new stent due to the involvement of the distal margin of the first. Interestingly, the patient did not report angina, although the absence of symptoms was most likely influenced by his limited physical activity due to the concomitant presence of a functionally limiting osteoarticular condition.

## 3 DISCUSSION

Stent underexpansion means a therapeutic challenge for interventional cardiologists nowadays. Although angiographic follow‐up studies supported by intracoronary imaging techniques and with sufficient sample sizes are required to assess the long‐term safety of IVL for the treatment of stent underexpansion, we believe that the case reported here may provide a very positive first indication on this issue, given the adverse clinical scenario presented and its good outcome despite the high intensity of the lithotripsy therapy used.

## AUTHOR CONTRIBUTIONS

Writing – original draft: José Valencia, Marta Herrero. Writing – review and editing: Juan Miguel Ruiz‐Nodar, Javier Pineda, Fernando Torres‐Mezcua. All authors have read and approved the final version of the manuscript. José Valencia had full access to all of the data in this study and takes complete responsibility for the integrity of the data and the accuracy of the data analysis.

## ACKNOWLEDGEMENT

The authors have received no financial support for this article.

## CONFLICT OF INTEREST

The authors declare no conflicts of interest.

## CONSENT

Written informed consent was obtained from the patient to publish this report in accordance with the journal's patient consent policy.

## TRANSPARENCY STATEMENT

The lead author (José Valencia) affirms that this manuscript is an honest, accurate, and transparent account of the study reported, that no important aspects of the study have been omitted, and that any discrepancies from the study as planned have been explained (and, if relevant, registered).

## Data Availability

Data sharing is not applicable to this article as no new data were created or analyzed in this clinical case report.
